# Effects of landscape metrics on scorpion (Arachnida: Scorpiones) assemblage in a tropical urban ecosystem

**DOI:** 10.1002/ece3.11026

**Published:** 2024-02-16

**Authors:** Matheus Leonydas Borba Feitosa, Hugo Rodrigo Barbosa‐da‐Silva, Renato Portela Salomão, Adriano Medeiros Desouza, Geraldo Jorge Barbosa de Moura, André Felipe de Araujo Lira

**Affiliations:** ^1^ Centro de Ciências Agrárias Universidade Federal da Paraíba Areia Paraíba Brazil; ^2^ Centro de Biociências Universidade Federal de Pernambuco Recife Pernambuco Brazil; ^3^ Facultad de Estudios Superiores Iztacala Universidad Nacional Autónoma de México Tlalnepantla de Baz Mexico; ^4^ Centro de Ciências Biológicas e da Saúde Universidade Estadual da Paraíba Campina Grande Paraíba Brazil; ^5^ Laboratorio de Estudos Herpetológicos e Paleoherpetológicos, Departamento de Biologia Universidade Federal Rural de Pernambuco Recife Pernambuco Brazil; ^6^ Colección Nacional de Arácnidos, Departamento de Zoología, Instituto de Biología Universidad Nacional Autónoma de México Ciudad de México Mexico

**Keywords:** arachnids, Buthidae, community ecology, habitat fragmentation, neotropical Forest

## Abstract

Urban landscapes restrain the distribution of forest‐dwelling species, which may be related to challenging conditions that impair body condition. The dynamics in urban areas lead to the simplification of communities that inhabit forest patches in cities with the turnover of sensitive species for opportunistic ones. In this study, we investigated the effect of urbanization on the body condition and diversity of scorpions at the landscape scale. Sampling was carried out in 10 forest patches in an urban matrix in Brazil, originally covered by a tropical rainforest. The surroundings of the landscape of each forest patch were characterized through the amount of forest, agriculture, and urban land cover. Individual body length, dry, lipid, and muscular masses were used as proxies of *Tityus pusillus* body condition. In total, 147 scorpions were collected, belonging to the species *Ananteris mauryi*, *T. pusillus*, *T. stigmurus*, and *T. neglectus*. Forest cover explained 28% of species variation. There was a positive relationship between forest cover and *T. pusillus* and *A. mauryi* abundances, while *T. stigmurus* was negatively affected by forest cover. Species richness and total scorpion abundance were not influenced by landscape metrics. In terms of body condition, only females of *T. pusillus* were affected by landscape variables, with individuals showing higher body mass with an increase in forest cover. Our results suggest that urban forests can support scorpion assemblages. However, there is a turnover in specialist forest species for opportunistic species. Forest cover is a crucial factor in maintaining healthy scorpion populations in urban areas.

## Graphical abstract

Urban landscapes restrain the distribution of forest‐dwelling species. Urban forests can support scorpion assemblages. However, there is a turnover in specialist forest species for opportunistic species

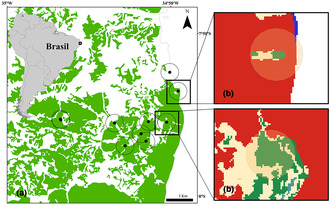

## 1 INTRODUCTION

Urbanization alters the landscape, transforming areas of native vegetation into industrial, agricultural, forestry, road networks, and residential areas (Adler & Tanner, 2015; Godron & Forman, 1983; Pickett & Rogers, 1997). Together with landscape changes, urbanization, which has impervious surfaces and buildings as its matrices, results in a decrease in green patches in cities (Fahrig, 2003; Forman & Godron, 1981, 1986; Sposito, 1988). There are marked effects of urbanization on ecological communities that inhabit and survive in cities, which are negatively impacted at the community and population scales (Fahrig & Merriam, 1985; Johnson & Munshi‐South, 2017; Salomão et al., 2019; White, 1985). With the decrease in the amount of native land cover, forest‐dweller species are more prone to the negative effects of the cities, such as urban waste inputs, competitive exclusion promoted by alien species, and abrupt shifts in microclimatic conditions (Du Toit et al., 2021; McKinney, 2002, 2008). Therefore, it is essential to disentangle the phenomenon of substitution of sensitive species for species that are more suitable when facing anthropogenic activities (“the winners and losers” paradigm, see Tabarelli et al., 2012). Therefore, the construction of urban ecosystems can be a controlling and filtering agent, which designates the survival of natural landscapes and acts as a selector of species suitable for urbanized conditions (Alberti, 2005; Alberti et al., 2003; Filgueiras et al., 2021; Fournier et al., 2020).

Species that inhabit urban regions or forest remnants near these regions experience additional and antagonistic stresses compared with those inhabiting natural habitats (Barber et al., 2010; Conti et al., 2004; Hope et al., 2022). Thus, understanding the effects on communities requires studies that aim to identify the impacts at the species level (Shochat et al., 2006). A fundamental aspect of urban ecology is identifying the nutritional status, physiological wear, and level of health of the animal, that is, its body condition (Schluter & Gustafsson, 1993). An organism's body condition can be easily targeted for negative implications subject to these pressures (Liker et al., 2008; Fenoglio et al., 2020; Salomão et al., 2019). A good body condition indicates that the skills and aptitudes of the organism are being tested naturally in regions where they are not subject to more difficulties (Jakob et al., 1996; Møller et al., 1998). It is possible to measure how fit the individual is to the environment by identifying the current level of the body condition of the specimen, thereby indicating the environmental stress to which he is subjected (Peig & Green, 2009, 2010; Thawley et al., 2019). Submission to prolonged stress can become harmful to the organism, inhibiting its growth, reproduction, physiology, and locomotion (Wingfield & Sapolsky, 2003).

Since the 16th century, Latin America has been a scenario of critical landscape transformation, focusing on providing natural resources for different regions of the world, primarily Europe and North America. For example, the establishment of Brazil resulted in non‐native colonization focused on the coastline. Currently, most Brazilian urban centers are distributed in this region (IBGE, 2022). Therefore, the biologically diverse Atlantic rainforest has undergone drastic forest loss dynamics (Brannstrom, 2002; Guedes et al., 2005; Myers et al., 2000). This alarming environmental transformation is accompanied by the establishment of a high number and density of urban centers, resulting in landscapes with city matrices in which forest remnants of different sizes and connectivity levels are distributed. Although previous studies suggest that the increase in urbanization in the Atlantic rainforest comes together with a decrease in species diversity (Enedino et al., 2017; Salomão et al., 2018, 2019; Silva‐Junior et al., 2018), few present mechanisms that connect the intraspecific conditions related to such biological impoverishment.

Among the organisms that inhabit tropical forests, arthropods have been used as models to measure human impact on natural communities (Menta & Remelli, 2020). Scorpions are habitat‐ and microhabitat‐specialized animals (Lira et al., 2018; Lourenço & Eickstedt, 2009; Polis, 1990). Therefore, the conditions necessary for their permanence change simultaneously as the ecosystems where they are located are altered (Laurance et al., 2007; Lira et al., 2016). Urban native forests, which are remnants of an urban matrix, can be more hostile than preserved forests (MacGregor‐Fors et al., 2021) and must express adverse conditions to withstand the assembly of scorpions. The availability of spatial and trophic resources is essential to achieve favorable body conditions that allow scorpions to maintain healthy populations (Jakob et al., 1996; Møller et al., 1998). Previous studies have observed that the alteration and conversion of the natural landscape to an urbanized matrix substantially reduces the richness and abundance of scorpions (Lira et al., 2019, 2021). Therefore, cues indicate that forest loss due to urbanization may impair scorpion diversity, which may be related to the reduction in population health conditions.

The objective of this study was to investigate the effect of landscape metrics on the assemblage (diversity and body condition) of scorpions in the urban remnants of the Atlantic rainforest, a highly urbanized and endangered tropical rainforest. We tested the following hypotheses: (I) an exchange of species will occur, favoring opportunistic species in more urbanized landscapes; (II) species richness, abundance, and body condition will be negatively affected by urbanization. To our knowledge, this is one of the first studies to incorporate assemblage diversity and body condition to assess the effects of anthropogenic disturbance. We believe that such research can expand our understanding of the effects of urbanization on species distribution and maintenance in urban remnants.

## 2 MATERIALS AND METHODS

### 2.1 Study area and landscape characterization

Fieldwork was carried out in 10 urban forest patches classified as seasonal semi‐deciduous, distributed in the municipality of Paulista, Pernambuco State, Brazil. The municipality of Paulista has a territorial area of 96,932 km^2^ and a population of ~336,919, presenting a population density of 3087 inhab/km^2^ (IBGE, 2022). Urban forest patches are scattered throughout the Paulista municipality and isolated by different types of anthropic matrices (such as urban settlements and sugarcane monoculture). Therefore, such forest patches served as natural laboratory to assess the response of native fauna to urbanization. Among the urban forest patches, 10 of them have a minimum level of security to allow a nocturnal fieldwork inside. The climate of the region is classified as Tropical Savanna (Aw/As) according to the Köppen classification, with an average annual temperature and precipitation of 25.6°C and 1121 mm, respectively (APAC, 2022).

**FIGURE 1:**
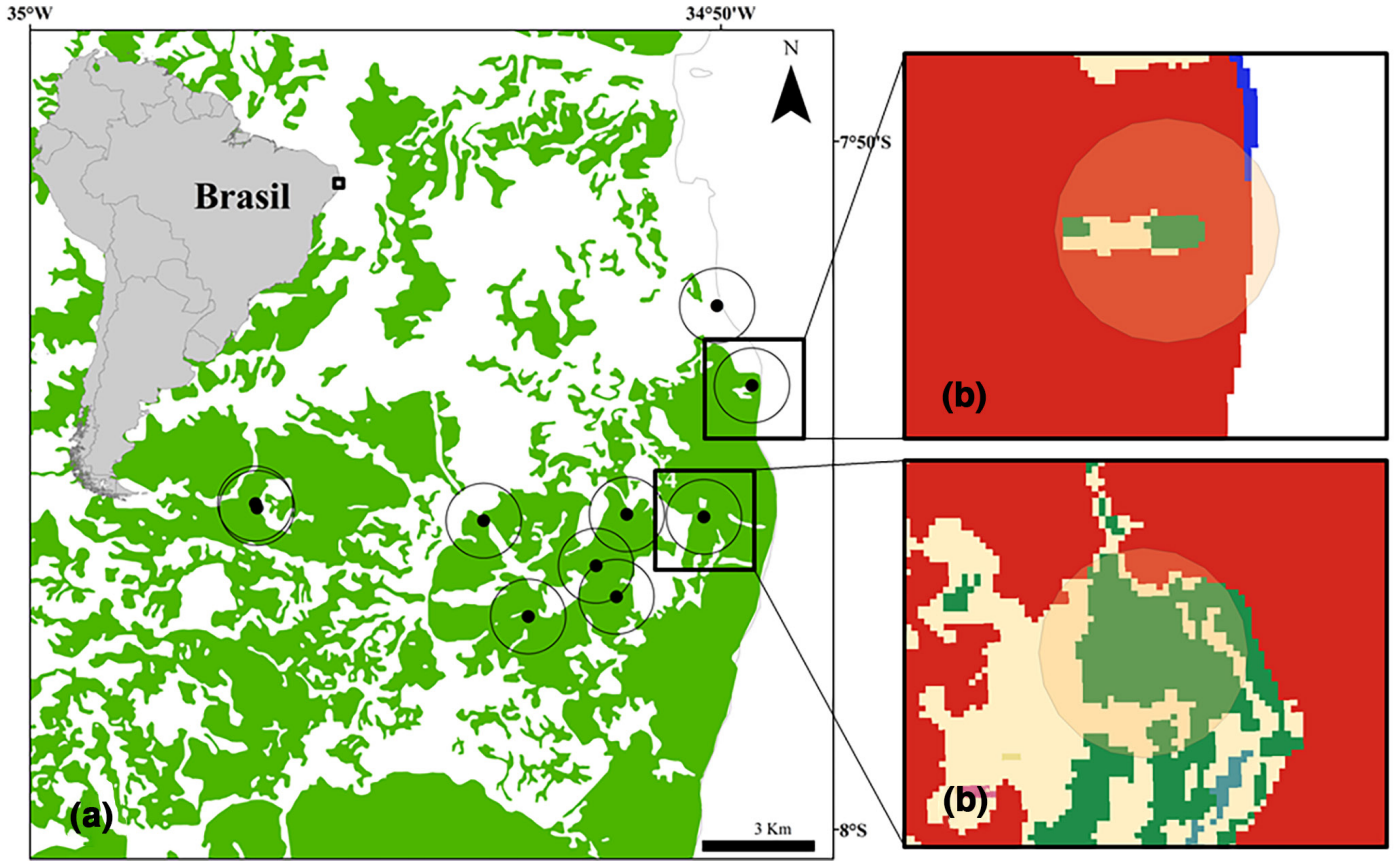
Distribution of scorpion collection sites in urban forest patches (a). Representation of the 500 m *buffer* around the collection site where the landscape metrics were calculated (b).

We used a landscape approach to analyze the potential effect of urbanization on scorpion assemblages. Landscape metrics included the area of forest cover, urban cover, and agricultural cover. Landscape characterization was performed using the percentage of metrics within a 500 m buffer around the urban forest patches to obtain the number of such land‐cover types (Figure 1). Land uses were extracted from the MapBiomas (Projeto MapBiomas, 2023) and quantified using the “zonal histogram” tool of the QGis 3.22 software (QGIS.org, 2022).

### 2.2 Scorpion sampling and body condition

Sampling was done during February 2022 through 1 h active nocturnal collections using ultraviolet flashlights between 19:00 and 22:00 h in each of the 10 urban forest patches studied. The collected individuals were stored in 70% ethanol for later identification, according to the key proposed by Lourenço (2002). After identification, measurements of the body condition of adult individuals were performed. The following parameters were used as proxies for the body condition: prosoma length, dry mass, lipid mass, and muscle mass. Prosoma length was measured using a digital caliper (0.01 mm). To obtain dry, lipid, and muscle mass, we followed the methodology described by Contreras‐Garduno et al. (2008) and González‐Tokman et al. (2011). Initially, the individuals were placed in an oven at 60°C for 48 h to remove all water from the animal and to obtain its body dry mass. The individuals were then placed individually in recipients containing chloroform for 24 h period for degrading their lipids. Subsequently, individuals were placed in an oven at 60°C for 48 h and re‐weighed. The mass obtained from the difference between the total dry mass and this new weight was considered the lipid mass. Scorpions were individually exposed to potassium hydroxide (0.8 mol/L) for 48 h to obtain muscle mass. Subsequently, they were washed in water, dried in an oven for 48 h at 60°C, and weighed again. The difference between the lipid mass and the new mass obtained was the muscle mass. Since body masses may be affected by individual body size, relative dry mass, relative lipid mass, and relative muscle mass were used in this study. Relative body masses of each individual were obtained by dividing individual body mass by its body size (i.e. carapace length).

### 2.3 Data analysis

The degree of collinearity between landscape metrics was determined using the Variance Inflation Factor (VIF) (Eisenlohr, 2014). VIF values greater than 10 indicate the multicollinearity of the data and decrease the strength of the analysis (Zuur et al., 2010). Thus, the urban cover was removed from our analysis (VIF > 10). Generalized linear models (GLMs) were used to assess the effect of landscape metrics (forest cover and agriculture cover) on scorpion species richness and abundance. For species richness, the data fitted the Poisson error distribution, whereas the abundance data best fitted the negative binomial distribution. The relationship between species composition and landscape metrics was determined using Redundancy Analysis (RDA). Its significance was calculated using 999 Monte Carlo permutations (Legendre & Legendre, 2012; Lepš & Šmilauer, 2003; McCune et al., 2002). Before RDA, a linear response of changes in species composition was confirmed using Detrended Correspondence Analysis (lengths of gradients = 2.41) (Lepš & Šmilauer, 2003).

Linear Models (LMs) were used to assess the effects of landscape metrics on scorpion body condition (prosoma size, relative dry mass, relative muscle mass, and relative lipid mass). Due to the limited statistical power to perform these models for most species (i.e. a reduced number of individuals per forest patch), only *T. pusillus* data was analyzed. This species was recorded in most of the forest patches and had a higher abundance (see Section 3). Since sex affected the distribution of scorpion body conditions among the study landscapes, we performed LMs separately for males and females.

GLMs and LMs were conducted using R software v. 4.1.3 (R Core Team, 2022). Negative binomial models were created using the mass packages (Ripley et al., 2022). Redundancy analysis was conducted using the CANOCO software v. 4.5 (ter Braak & Smilauer, 2002).

## 3 RESULTS

In total, 147 scorpions of the Buthidae family were collected and represented by four species. In each urban forest patch, the number of species ranged from one to three, with *Tityus pusillus* being the most abundant and widely distributed species, representing 70% of the total abundance. It was distributed in seven fragments (Table 1). *Tityus stigmurus* (Thorell 1876) and *Ananteris mauryi* Lourenço 1982 represented 29.25% of the scorpions and were sampled in four and five fragments, respectively (Table 1). *Tityus neglectus* was represented by a single individual.

**TABLE 1:** Scorpion diversity from 10 urban forest patches in Paulista municipality, Brazil.

Urban forest patch	Scorpion species				Total
	*Ananteris mauryi*	*Tityus neglectus*	*Tityus pusillus*	*Tityus stigmurus*	
1	7	0	16	1	24
2	0	0	0	1	1
3	3	0	3	0	6
4	3	0	31	0	34
5	0	1	4	0	5
6	0	0	6	0	6
7	1	0	12	2	15
8	2	0	31	0	33
9	0	0	0	23	23
10	0	0	0	0	0

**FIGURE 2:**
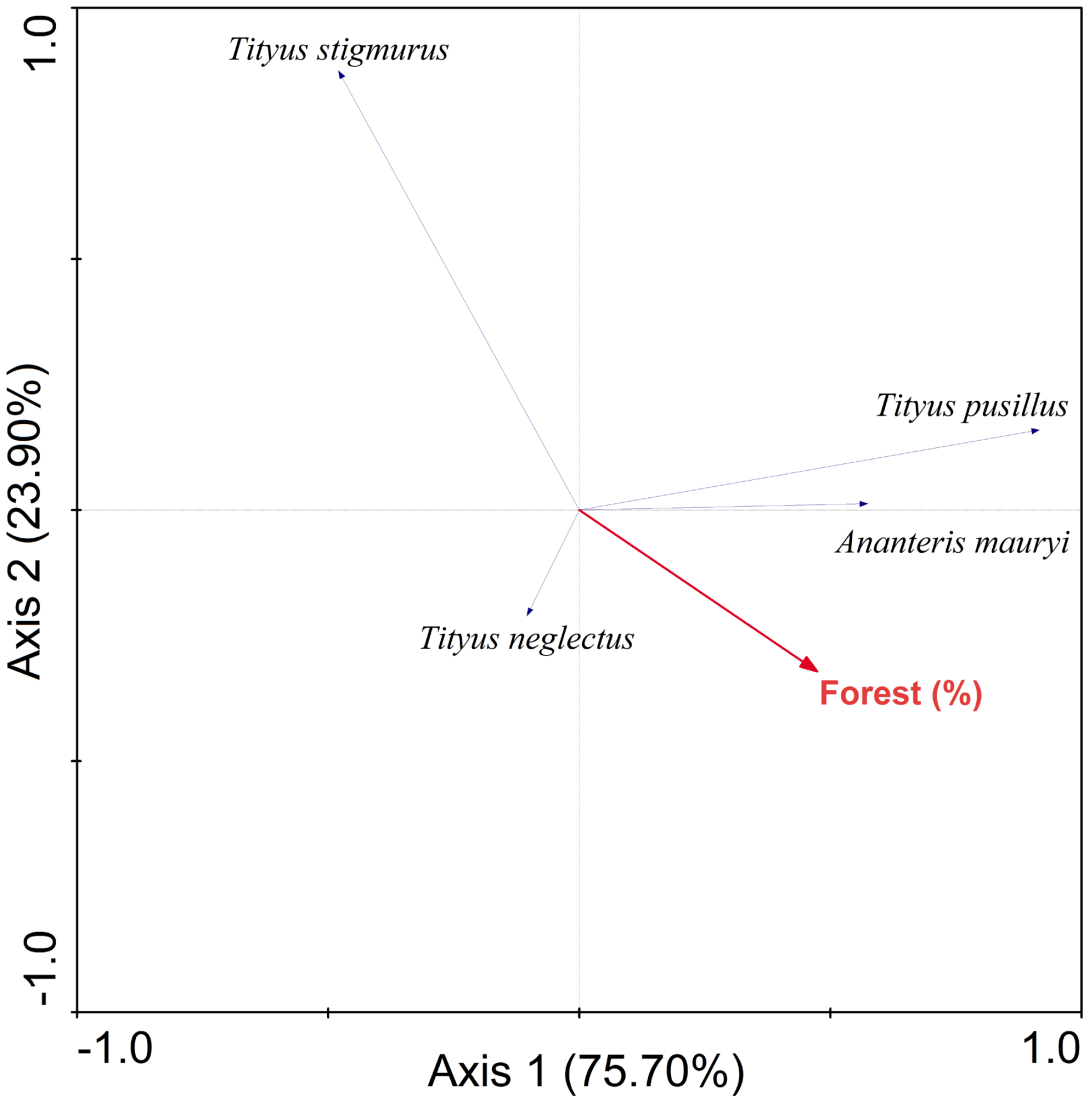
Redundancy analysis (RDA) ordination diagram of the species composition of the scorpion assemblage in relation to the forest cover in 10 urban forest patches.

The amount of the different land cover types did not affect species richness (forest cover: *F*_1,7_ = 2.56, *p* = .76; monoculture: *F*_1,6_ = 1.80, *p* = .38) or abundance (forest cover: *F*_1,7_ = 11.40, *p* = .95; monoculture: *F*_1,6_ = 9.80, *p* = .20). However, landscape metrics explained 99.6% of the species variation of which 75.7% was explained by axis 1 and 23.9% by axis 2 of the RDA. Forest cover was the only metric related to scorpion assemblage (RDA: *F* = 5.78; *p* = .04), explaining 28% of the species variation. Scorpions showed different responses to forest cover, with the species *T. pusillus* and *A. mauryi* being positively related to this metric, while *T. stigmurus* was negatively related (Figure 2).

The body condition of *T. pusillus* individuals was affected exclusively by forest cover and its response was sex‐dependent (Table 2). Urban forest fragments with higher amounts of forest cover presented females with higher body dry mass and lipid mass than fragments with lower amounts of forest cover. The other landscape metrics did not affect the body condition of females. Furthermore, male body condition was unaffected by landscape parameters (Table 2).

**TABLE 2:** Linear Models were used to test the effect of landscape metrics on the body condition of *Tityus pusillus* scorpions in urban forest patches.

Landscape metrics	Males		Females	
	*F*	*p*	*F*	*p*
Forest cover				
Prosoma length	0.02	.88	1.18	.28
Relative dry mass	0.00	.95	4.69	**.03 (+)**
Relative lipid mass	0.45	.50	5.30	**.02 (+)**
Relative muscle mass	0.35	.55	1.10	.29
Monoculture				
Prossoma length	0	.99	3.13	.08
Relative dry mass	1.01	.32	0.39	.53
Relative lipid mass	0.34	.56	0.25	.61
Relative muscle mass	0.80	.37	0.77	.38

*Note*: Statistically significant relationships are shown in bold; “+” = positive relationship.

## 4 DISCUSSION

In this study, we evaluated the effects of landscape structure on scorpion assemblages in urban forest patches located in the most urbanized tropical rainforests of Brazil. Our results revealed that landscape structure shaped the scorpion species composition. In addition, body condition was affected by forest cover in females and not males of the most abundant scorpion species. Our data corroborate previous studies that indicate that urban forest patches can maintain wild fauna (Magura et al., 2010; Start et al., 2020), but there is a physiological cost for the species that endure such patches. When compared with previous studies in non‐urban matrices located in tropical rainforests (e.g., Dias et al., 2006; Lira et al., 2016, 2019), scorpion species richness tended to be higher than that in the urban landscape. Through the combination of taxonomic diversity and body condition, we presented cues indicating that challenging scenarios for the maintenance of scorpion species can be observed in urbanized landscapes.

The assemblage composition of scorpions was influenced by landscape structure and linked to forest cover. Our results showed that species exchange was related to the percentage of forest cover around the sampled forest patch. Species typical of forested areas, such as *T. pusillus* and *A. mauryi* (Lira et al., 2020), were positively correlated with forest cover. However, the decrease in forest cover favored the presence of the habitat‐generalist scorpion *T. stigmurus*. This species is synanthropic, with a greater abundance in urban regions than in natural ones (Amado et al., 2021; Foerster et al., 2021, 2022). Previous studies indicated that Atlantic Forest scorpion assemblages are highly sensitive to changes in habitat at a local or landscape scale (Lira et al., 2016, 2020, 2021). Therefore, environmental variables such as the percentage of vegetation in urban forests can act as environmental filters that influence and select species (Filgueiras et al., 2011; Uehara‐Prado et al., 2007). Thus, the reduction in forest cover may favor the permanence of habitat‐generalist species to the detriment of habitat‐specialist species.

Forest cover was also a determining factor for the body condition of *T. pusillus* populations. Only females were responsive to landscape metrics presenting higher dry and lipid masses in patches with higher forest cover. The lipid mass is described as a source of energy material, and the dry mass corresponds to the animal's biomass, both obtained from the diet (Drewnowski, 1992; Wymann & Schneiter, 2008). Therefore, the low values of dry and lipid masses related to less forested regions may be associated to females exhibiting considerably less active foraging behaviors than males (Lira et al., 2018). This leads to a diet with longer time intervals or connected to food sources of lower nutritional value due to their residence in the region. Thus, urbanization may negatively impact the *T. pusillus* population by reducing female fitness. A previous study found that *T. pusillus* females subjected to dietary restrictions had a higher mortality rate, prolonged gestational period, and produced weak litters than those without dietary restrictions (Silva‐Júnior et al., 2022). Thus, the body condition of scorpions is shaped by the effect of urbanization during the growth of the individual (Olivero et al., 2021). Although scorpions are considered generalist predators, our data suggest that populations of *T. pusillus* residing in areas with low forest cover are maintained under suboptimal conditions.

## 5 CONCLUSIONS

In summary, by analyzing the correlation of landscape structure with the assemblage of scorpions in forest fragments in urban areas, our study showed the magnitude of the effect of urbanization on these arachnids. Although scorpion richness and abundance were not affected by any landscape metric used in this study, we found that the role of vegetation was crucial in species composition. According to our results, the habitat‐specialist species *T. pusillus* and *A. mauryi* were replaced by the habitat‐generalist species *T. stigmurus* as forest cover was reduced. The body condition of *T. pusillus* females was elusive through parameters related to the acquisition of food resources, such as lipid and dry masses and vegetation, with individuals from less forested areas presenting lower values.

## AUTHOR CONTRIBUTIONS

**Matheus Leonydas Borba Feitosa:** Data curation (equal); investigation (equal); methodology (equal); writing – original draft (equal). **Hugo Rodrigo Barbosa‐da‐Silva:** Data curation (equal); investigation (equal); methodology (equal); writing – original draft (equal). **Renato Portela Salomão:** Conceptualization (equal); formal analysis (equal); methodology (equal); validation (equal); writing – review and editing (equal). **Adriano Medeiros Desouza:** Formal analysis (equal); investigation (equal); methodology (equal); writing – review and editing (equal). **Geraldo Jorge Barbosa de Moura:** Conceptualization (equal); funding acquisition (equal); project administration (equal); resources (equal); writing – review and editing (equal). **André Felipe de Araujo Lira:** Conceptualization (equal); data curation (equal); formal analysis (equal); methodology (equal); supervision (equal); validation (equal); writing – review and editing (equal).

## FUNDING INFORMATION

AFAL was supported by Dirección General de Asuntos del Personal Académico (DGAPA) postdoctoral fellowship from the Universidad Nacional Autónoma de México. MLBF was supported by Coordenação de Aperfeiçoamento de Pessoal de Nível Superior (CAPES) for a postgraduate scholarship.

## CONFLICT OF INTEREST STATEMENT

The authors declare that they have no conflict of interest.

## Supporting information


Data S1:

## Data Availability

The dataset used in this study is available in supplementary material.
